# Depression-/Anxiety-Like Behavior Alterations in Adult Slit2 Transgenic Mice

**DOI:** 10.3389/fnbeh.2020.622257

**Published:** 2021-02-05

**Authors:** Guilan Huang, Sheng Wang, Jie Yan, Changxi Li, Jianwen Feng, Qi Chen, Xiaomeng Zheng, Huimin Li, Yajun He, Andrew J. Young, Haobin Li, Weidong Li, Jiangchao Li, Lijing Wang

**Affiliations:** ^1^School of Nursing, Guangdong Pharmaceutical University, Guangzhou, China; ^2^School of Life Sciences and Biopharmaceuticals, Guangdong Pharmaceutical University, Guangzhou, China; ^3^School of Pharmacy, Guangdong Pharmaceutical University, Guangzhou, China; ^4^School of Clinical Medicine, Guangdong Pharmaceutical University, Guangzhou, China; ^5^Department of Applied Psychology, School of Humanities and Communication, Guangdong University of Finance & Economics, Guangzhou, China; ^6^Department of Pathology, Bao'an People's Hospital of Shenzhen, Guangdong, China; ^7^Columbus Inpatient Care, Grove City, OH, United States; ^8^School of Health, Guangdong Pharmaceutical University, Guangzhou, China

**Keywords:** Slit2, transgenic mice, depression, anxiety, behavior

## Abstract

**Background:** Slit2 is a member of the Slit family of secreted glycoproteins that plays highly conserved roles in neuronal axon guidance and cellular migration. Our previous experimental results showed Alzheimer's disease-like alterations and increased permeability of the blood–brain barrier in Slit2-overexpressing transgenic (Slit2-Tg) mice aged 8–9 months. Nevertheless, relatively little is known about behavioral alterations in adult Slit2-Tg mice (2–6 months of age). To observe the age-related behavioral effects of Slit2 overexpression in adult mice, we performed a battery of behavioral tests with adult Slit2-Tg mice at 2–6 months of age.

**Results:** The body weight of Slit2-Tg mice was lower than that of the wild-type mice from 15 weeks of age. Compared with the control mice, depression-like behaviors were found in Slit2-Tg mice from 15 to 21 weeks of age in the sucrose preference test, although Slit2-Tg mice were hyperactive in the tail suspension test. The anxiety-like behaviors were found in Slit2-Tg mice in the open field test, as well as increased locomotor activity. The anxiety-like behaviors were also found in adult Slit2-Tg mice in the elevated plus maze. Compared to wild-type mice at 23 weeks old, impairment of the hippocampal neurons were found in Slit2-Tg mice at the same age in hematoxylin–eosin staining (H&E), including some eccentric dispersion and expansion of neuronal bodies. In addition, the messenger RNA (mRNA) expression of TNF-α was elevated in the hippocampus of adult Slit2-Tg mice.

**Conclusions:** Slit2 overexpression causes depression-/anxiety-like behaviors in adult mice that may be related to an increase in inflammatory factors and damage to hippocampal neurons.

## Introduction

Slit2 is an extracellular matrix protein that is essential for central nervous system (CNS) development (Bagri et al., 2002). Slit2 is synthesized in midline glial cells and expressed in neurons in the CNS. Slit2 is also expressed in the liver, mammary gland, kidney, heart, and lung and modulates branch formation of sensory axons from the dorsal root ganglia by binding to their transmembrane receptor, Roundabout (Robo) (Ballard and Hinck, 2012; Chaturvedi and Robinson, 2015; Tong et al., 2019). Slit/Robo signaling directs the migration of multiple neuron types and regulates the proliferation of central nervous system progenitor cells (Kidd et al., 1999; Borrell et al., 2012). In addition, Slit2 participates in the growth of neurites and the regulation of neuron branches and guides axons to form synaptic clues (Andrews et al., 2006; Ke et al., 2017). In summary, Slit2 plays a highly conserved role in CNS development especially in axonal navigation and cellular migration. Slit/Robo signaling also mediates other biological processes such as angiogenesis, leukocyte chemotaxis, cell proliferation, and tumor growth and progression (Borrell et al., 2012; Yuen and Robinson, 2013; Jiang et al., 2019). Increasing evidence suggests that disturbance in Slit/Robo signaling plays a role in several neuropsychiatric disorders, including Alzheimer's disease (AD), autism spectrum disorder (ASD), temporal lobe epilepsy (TLE), and major depressive disorder (MDD) (Fang et al., 2010; Li et al., 2015; Gorker et al., 2018; Bai et al., 2020). Bai et al. found that the Slit2 protein could be a novel biomarker candidate through proteomics profiling of the cerebrospinal fluid (CSF) in patients with AD (Bai et al., 2020). Our previous study found that the permeability of the blood–brain barrier (BBB) was increased, and the survival of hippocampal neurons was diminished in Slit2 transgenic mice aged 8–9 months. Moreover, Slit2 transgenic mice exhibited amyloid-β (Aβ) protein deposition and impaired leaning and cognitive function (Li et al., 2015). However, the changes in Slit2-overexpressing mice aged 2–6 months are not fully understood. A comprehensive understanding of the effects of Slit2 overexpression on the behavior and brain function of mice may clarify the role of Slit2 in some neuropsychiatric disorders.

Depression and anxiety are common mental disorders in the adult population. More than 350 million people worldwide suffer from depression, and the overall prevalence is ~6% (Lepine and Briley, 2011; Kessler and Bromet, 2013). Depression has become the main cause of disability worldwide and global disease burden (Smith, 2014). At present, the global prevalence of anxiety disorders is 7.3%, and the incidence of comorbidities with other mental diseases is 7–32% (Yerevanian et al., 2001; Stein et al., 2017). In addition, most studies focus on comorbidity between depression and anxiety disorders (Preti et al., 2016). Genetic vulnerability and environmental stressors are the two broad causes of psychiatric disorders such as depression and anxiety (Heim and Binder, 2012; Smoller, 2016). However, the pathophysiology of anxiety and depression disorder currently remains elusive. Therefore, their pathogenesis has become a key research question that needs to be urgently solved. Animal models of psychiatric disorders are widely used in preclinical research on mental disorders (Scherma et al., 2019). Chronic unpredictable mild stress (CUMS) is the most commonly used rodent model of depression. However, this method requires a significant and tedious workload, a long modeling time, and a large amount of manpower and material resources and has poor repeatability. Studies have shown that mice have better genetic possibilities than rats (El Yacoubi and Vaugeois, 2007). Choosing the most suitable animal model determines the success or failure of the experiment and the scientific and objective nature of the research.

In this study, we analyzed the behavior of Slit2-Tg mice at the age of 2–6 months via a battery of behavioral tests, aiming to investigate whether the overexpression of Slit2 protein cause behavioral changes in adult mice. The results indicate important roles of Slit2 in brain functions and the potential of Slit2-Tg mice as a model of spontaneous depression and anxiety.

## Materials and Methods

### Animals

Eight-week-old male C57BL/6J mice, weighing 22–25 g were obtained from Guangdong Animal Centre (Guangzhou, China). In addition, 8-week-old Slit2 overexpression transgenic mice (Slit2-Tg mice, C57BL/6 background) weighing 22–25 g were generated by the institute of Biochemistry and Cell Biology (CAS, Shanghai, China), as previously described (Han and Geng, 2011). Thirty Slit2-Tg male mice (9 weeks old, *n* = 30; 15 weeks old, *n* = 20; 21 weeks old, *n* = 10) and 30 wild-type control (C57BL/6J) male mice (9 weeks old, *n* = 30; 15 weeks old, *n* = 20; 21 weeks old, *n* = 10) were used in this study. All mice were housed five per cage (260 × 160 × 128 mm) under 50–60% humidity, controlled temperature (22–26°C), and a 12-h light/dark cycle (lights on at 7:00 am). The cage is cleaned every 5 days. They were allowed free access to water and food and acclimatized for 1 week before use. All experimental procedures were approved by the Animal Ethics Committee of Guangdong Pharmaceutical University.

### Weight and Behavioral Tests

Behavioral tests were performed for each mouse between 8:00 a.m. and 7 p.m. on weeks 9 (*n* = 60), 15 (*n* = 40), and 21 (*n* = 20) during the growth of the mice. During this period, each mouse was weighted once per week. The mice were tested in the following order: weight, sucrose preference test, open field test, elevated plus maze test, tail suspension test, and forced swimming test. Following the behavioral test, the mice were fasted for 12 h. Then, they were sacrificed by decapitation (Supplementary Figure 1). The testing apparatus was cleaned after the test. The interval time between behavioral tests was at least 24 h.

### Sucrose Preference Test

The sucrose preference test was conducted as described in Willner et al. with minor modifications (Willner et al., 1987; Song et al., 2018). Every cage was given two bottles containing 1% sucrose solution for 24 h adaptation; then, the sucrose in one bottle was replaced with pure water for the next 24 h before the test. After adaptation, each mouse was deprived of water and food for 24 h. Then, each animal was given free access to two bottles containing pure water or 1% sucrose solution for 24 h. To avoid bottle side preference, we changed the position of the two bottles after 12 h. Sucrose preferences were calculated according to the following formula: sucrose preference (%) = sucrose consumption/(water consumption + sucrose consumption) × 100%.

### Open Field Test

The open field test was used to assess the locomotor activity and anxiety-like behavior, as previously described (Rodrigues et al., 1996; Liu et al., 2019). Mice were assessed in a box measuring 50 × 50 × 18.5 cm. After 2 days of adaptation to the device, each mouse was placed in the box and allowed to explore freely for 5 min. A video camera recorded the data, including total duration of travel in the center zone (s), the percentage of distance traveled in the center (%), number of crossings, vertical activity, and total distance (cm), and the data were analyzed by software (SuperMaze Software, XingRuan Shanghai, China). The bottom and inner walls of the box were cleaned with 75% ethanol between tests.

### Elevated Plus Maze Test

The elevated plus maze test was used to evaluate anxiety-related behavior, as previously described (Lister, 1987; Komada et al., 2008). The elevated plus maze consisted of two closed arms (width × length × wall height: 6 × 40 × 15 cm) and two open arms and was elevated 45 cm above the floor. Each mouse was placed at the center of the EPM (facing the open arm) and allowed to explore for 5 min. Distance traveled in the open arms (%), time spent in the open arms (%), time spent in the closed arms (s), entries into the open arms (%), and distance traveled (cm) were recorded using software (SuperMaze Software, XingRuan Shanghai, China). Before each test began, the experimental apparatus was cleaned with 75% ethanol.

### Tail Suspension Test

The tail suspension test was used to assess depression-related behavior (Steru et al., 1985; Xian et al., 2019). Mice were suspended 15 cm above the floor using an adhesive tape for 6 min. After 1 min of adaptation, the total duration of immobility over the next 5 min was calculated. If they hung down completely motionless, the mice were considered immobile. The total immobility time (s) was recorded during the last 5 min of a 6-min test session by two observers who were blinded to the group's conditions.

### Forced Swimming Test

The forced swimming test was conducted as previously described (Porsolt et al., 1977; Xian et al., 2019). The mice were individually forced to swim in a transparent beaker (diameter, 13.3 cm; height, 19.8 cm) filled with 13 cm water (24–26°C) for 6 min. The total immobility time (s) was recorded during the last 5 min of a 6-min test session by two observers who were blinded to the group's conditions. Mice were considered immobile whenever they were floating with only slight movements necessary to keep afloat. The water was changed for each mice.

## Biochemical Analysis

### Tissue and Blood Sampling

Following the behavioral test, the mice were fasted for 12 h. Then, they were anesthetized with ether, and blood was collected. After coagulation for 1 h, blood samples were centrifuged at 5,000 rpm and 4°C for 30 min. Finally, the serum was collected and stored in a −80°C refrigerator until use. The mice were transcardially perfused with phosphate-buffered saline (PBS), followed by 4% paraformaldehyde. The whole brains were removed and immersed in 4% paraformaldehyde for H&E staining. After the remaining mice were sacrificed by cervical dislocation, the hippocampal tissue was dissected in ice-cold PBS and then quickly stored at −80°C until use.

### HE Staining/Histopathological Analysis of the Hippocampus

Hematoxylin–eosin staining (H&E) was used to reveal the histopathological features of the mouse hippocampus. Whole brains were removed from mice and immersed in 4% paraformaldehyde for 24 h followed by running water for 8 h. After dehydration, the whole brains were processed for paraffin embedding and cut into 4-μm thick coronal sections. Subsequently, paraffin sections of the hippocampus were deparaffinized with xylene and stained with hematoxylin and eosin following the manufacturer's instructions. Next, images of the hippocampus were collected using a light microscope. As shown in Figure 4, the CA2 region was indicated with a block border.

### RNA Extraction and qRT-PCR

RNA was extracted from the hippocampus of mouse brains using Trizol reagent (TaKaRa, Japan, Code: 9109) and synthesized into complementary DNA (cDNA) using an Evo M-MLV Reverse Transcription kit (AG, China, Code:AG11711) following the reagent instructions. The cDNA for glyceraldehyde 3-phosphate dehydrogenase (GAPDH), BDNF, 5-hydroxytryptamine receptor 1A (Htr1a), nuclear receptor subfamily 3 group C member 1 (NR3C1), TNF-α, IL-6, and IL-1β was amplified by PCR with specific primers and assayed by a LightCycler ®96 Real-Time PCR Detection System (Roche, Basel, Switzerland). Gene expression was analyzed using the 2^−ΔΔ*Ct*^ method. Levels of targeted messenger RNA (mRNA) were normalized to the GAPDH. The primer sets are shown in Table 1.

**Table 1:** Specific primers sequences of target genes (RT-PCR Primers).

**Mouse gene/target**	**Primer sequence (**5^′^** to **3^′^)****
IL-1β	Forward: TCGCAGCAGCACATCAACAAGAG
	Reverse: AGGTCCACGGGAAAGACACAGG
IL-6	Forward: TAGTCCTTCCTACCCCAATTTCC
	Reverse: TTGGTCCTTAGCCACTCCTTC
TNF-α	Forward: AGACAGAGGCAACCTGACCAC
	Reverse: GCACCACCATCAAGGACTCAA
BDNF	Forward: CCCATGAAAGAAGTAAACGTCC
	Reverse: CCTTATGGTTTTCTTCGTTGGG
NR3C1	Forward: GGCAGCGGTTTTATCAACTG
	Reverse: TCAGCTAACATCTCTGGGAATTC
Htr1a	Forward: AATGTTGCCAACTATCTCATCG
	Reverse: GTTCACGTAGTCTATAGGGTCG
GAPDH	Forward: CGTCCCGTAGACAAAATGGT
	Reverse: TCAATGAAGGGGTCGTTGAT

### Immunohistochemistry

Paraffin sections of the hippocampus were deparaffinized with xylene and washed with PBS. Next, antigen repair was performed in sodium citrate buffer (pH 6.0) with pressure for 20 min and then permeabilized with methanol containing 3% hydrogen peroxide for 30 min. Subsequently, the paraffin sections were incubated with 10% bovine serum albumin (BSA) at 37°C for 1 h. Then, the sections were incubated with rabbit anti-Slit2 antibody (1:50, Proteintech, USA, catalog number: 20217-1-AP) overnight at 4°C. The next day, after washing, the paraffin sections were incubated with goat antirabbit secondary antibody (1:100, Zhongshan Golden Bridge, China) at 37°C for 1 h and stained with 3′, 3′-diaminobenzidine-tetrahydrochloride (DAB) for 1 min.

### Statistical Analysis

All data are expressed as the mean ± SEM and were analyzed using SPSS 26.0 (IBM SPSS, USA) and Prism 8 (GraphPad Software, USA). Unpaired *t*-test or two-way analysis of variance was used to test for statistical significance. *P* < 0.05 was considered statistically significant. Bars represent the mean ± SEM.

## Results

### Adult Slit2-Tg Mice Exhibited Depression-Like Behavior

**Figure 1:**
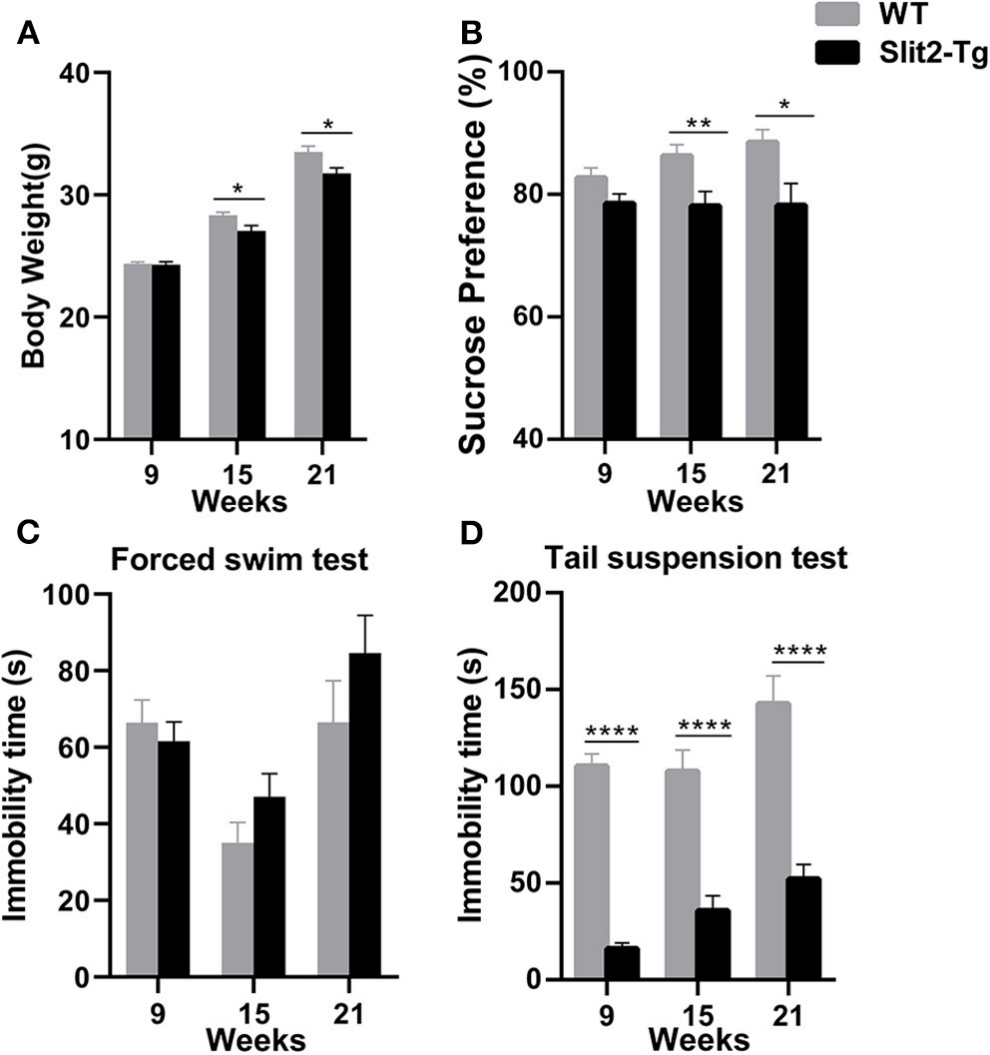
Depression-like behavior of Slit2-Tg mice. **(A)** Twenty-one-week-old Slit2-Tg mice exhibited reduced body weight compared with wild-type (WT) mice at the same age. **(B)** Sucrose preference test showing a decreased sucrose preference of Slit2-Tg mice compared with WT mice. **(C)** Forced swimming test showing an increased duration of immobility of 21-week-old Slit2-Tg mice compared with WT mice at the same age. **(D)** Tail suspension test showing a decreased immobility time of Slit2-Tg mice compared with WT mice. All data are expressed as means ± standard error of the mean (SEM), *p < 0.05, **p < 0.01, ****p < 0.0001 vs. WT mice (Slit2-Tg mice: 9 weeks, *n* = 30; 15 weeks, *n* = 20; 21 weeks, *n* = 10; WT mice: 9 weeks, *n* = 30; 15 weeks, *n* = 20; 21 weeks, *n* = 10).

Decreased weight and sucrose preference were discovered in adult Slit2-Tg mice as well as increased immobility time of adult Slit2-Tg mice in the forced swim test. We regularly observed the changes in the weights of the mice. As shown in Figure 1A, from the age of 15 weeks, the Slit2-Tg mice exhibited significantly lower body weight than wild-type (WT) mice [weight at 15 weeks: *t*(38) = 2.636, *p* = 0.0121; weight at 21 weeks: *t*(18) = 2.647, *p* = 0.0164]. The sucrose preference test by each group was significantly affected by genotype (two-way ANOVA: *F*_1, 114_ = 17.299, *p* < 0.0001) but not by time (*F*_2, 114_ = 0.847, *p* = 0.431), and the genotype × time interaction was not significant (*F*_2, 114_ = 1.121, *p* = 0.330). From the age of 15 weeks, Slit2-Tg mice showed anhedonia, as the sucrose preference index was lower than in mice in the sucrose preference test, indicating depression-like behavior in 21-week-old Slit2-Tg mice [15 weeks: *t*(38) = 2.764, *p* = 0.0088; 21 weeks: *t*(18) = 2.527, *p* = 0.0211, Figure 1B]. However, our results showed that the sucrose preference index was not significantly different between Slit2-Tg and WT mice at 9 weeks [9 weeks: *t*(58) = 0.6860, *p* = 0.4954, Figure 1B].

Depression-like behaviors in Slit2-Tg mice were also examined in the forced swim test and tail suspension test. In the forced swim test, the immobility time was significantly affected by time (two-way ANOVA: *F*_2, 114_ = 11.778, *p* < 0.0001) but not by genotype (*F*_1, 114_ = 2.090, *p* = 0.151), and the genotype × time interaction was not significant (*F*_2, 114_ = 1.675, *p* = 0.192). As shown in Figure 1C, 21-week-old Slit2-Tg mice exhibited depression-like behavior with a longer immobility time than 21-week-old WT mice in the forced swim test, although there was no significant difference [*t*(18) = 1.242, *p* = 0.2301, Figure 1C]. No obvious difference in immobility time was found in the forced swim test between Slit2-Tg and WT mice at 9 or 15 weeks [9 weeks: *t*(58) = 0.6268, *p* = 0.5333; 15 weeks: *t*(38) = 1.503, *p* = 0.1412]. However, in the tail suspension test, the immobility time was affected by time (two-way ANOVA: *F*_2, 114_ = 7.320, *p* = 0.001) and genotype (*F*_1, 114_ = 150.484, *p* < 0.0001), with no significant genotype × time interaction (*F*_2, 114_ = 1.306, *p* = 0.275). Slit2-Tg mice consistently spent less time immobile than WT mice [9 weeks: *t*(58) = 13.25, *p* < 0.0001; 15 weeks: *t*(38) = 5.338, *p* < 0.0001; 21 weeks: *t*(18) = 5.629, *p* < 0.0001, Figure 1D].

### Increased Anxiety-Like and Locomotor Activity of Slit2-Tg Mice in the Open Field Test

**Figure 2:**
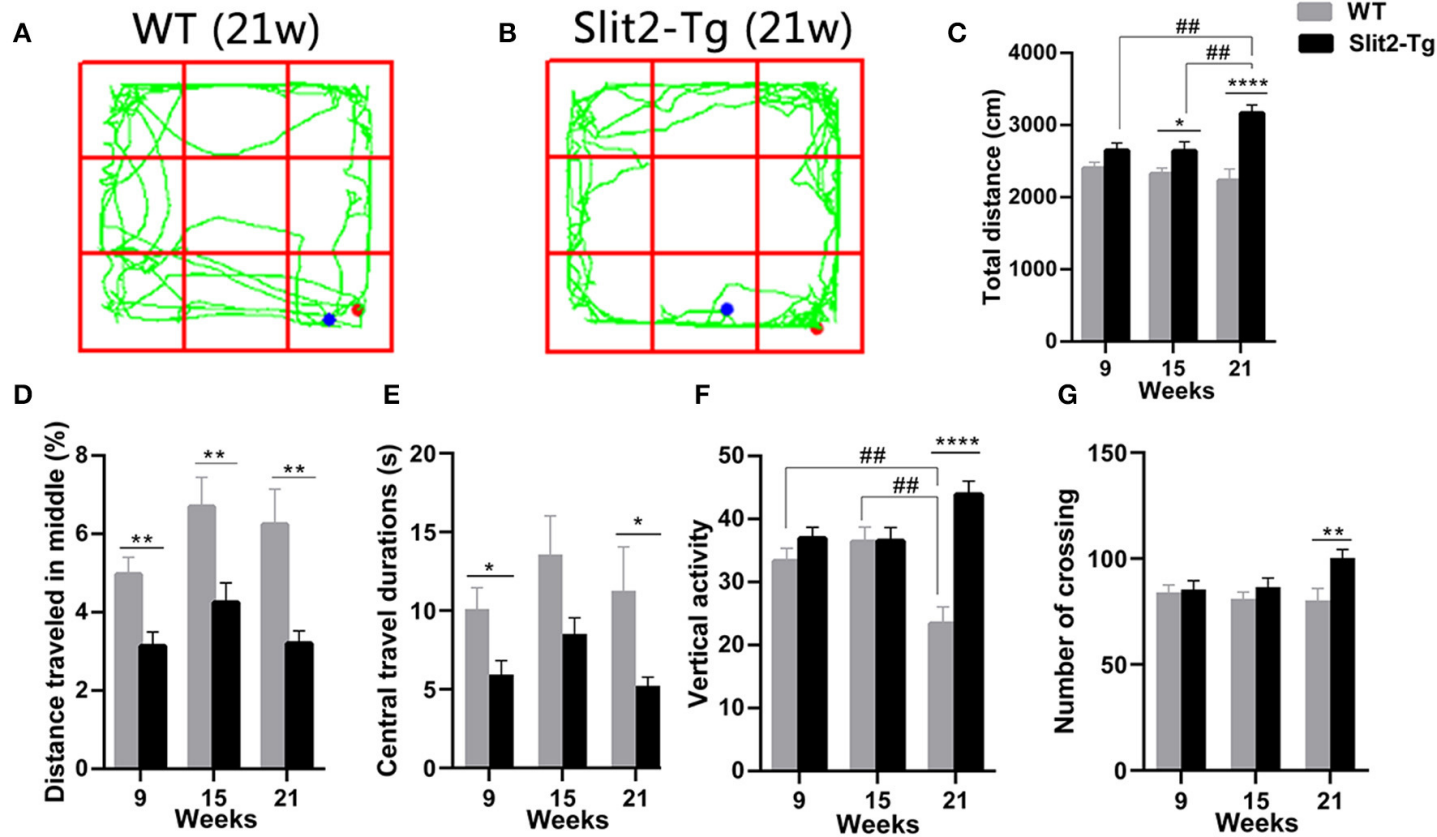
Increased locomotor activity and anxiety-like behavior of Slit2-Tg mice observed in the open field test. **(A,B)** show the trajectories of mice in the open field test. **(C)** Total distance traveled. **(D)** The percentage of distance traveled in the middle. **(E)** Central travel durations: Slit2-Tg mice showed shorter central travel durations than wild-type (WT) mice. **(F)** Vertical activity. **(G)** Number of crossings. All data are expressed as means ± SEM. *p < 0.05, **p < 0.01, ****p < 0.0001 vs. WT mice. ^##^*p* < 0.01 (Slit2-Tg mice: 9 weeks, *n* = 30; 15 weeks, *n* = 20; 21 weeks, *n* = 10; WT mice: 9 weeks, *n* = 30; 15 weeks, *n* = 20; 21 weeks, *n* = 10).

We used a 5-min open field test to further evaluate the anxiety- or depression-like behaviors of Slit2-Tg mice. We found that the duration of time spent at the center of the open field was significantly affected only by genotype (two-way ANOVA: genotype: *F*_1, 114_ = 12.855, *p* < 0.0001; time: *F*_2, 114_ = 2.387, *p* = 0.096; genotype × time interaction: *F*_2, 114_ = 0.146, *p* = 0.864). The percentage of distance traveled at the center was significantly affected by genotype (*F*_1, 114_ = 24.941, *p* < 0.0001) and time (*F*_2, 114_ = 4.168, *p* = 0.018), with no significant genotype × time interaction (*F*_2, 114_ = 0.522, *p* = 0.594). As shown in Figure 2, in the 9-week-old mice, the duration of time spent at the center [*t*(58) = 2.582, *p* = 0.0124, Figure 2E] and the percentage of distance traveled at the center of the open field [*t*(58) = 3.218, *p* = 0.0021, Figure 2D] were significantly lower in Slit2-Tg mice than in WT mice, indicating increased anxiety-like behavior in 9-week-old Slit2-Tg mice. In the 15-week-old mice, the percentage of distance traveled at the center [*t*(38) = 2.739, *p* = 0.0093, Figure 2D] was significantly lower in Slit2-Tg mice than in WT mice. In addition, the duration of time spent at the center of the open field in the 15-week-old Slit2-Tg mice tended to decrease compared with WT mice at the same age [*t*(38) = 1.914, *p* = 0.0632, Figure 2E]. Surprisingly, the 15-week-old Slit2-Tg mice exhibited increased locomotor activity, as the total distance traveled exceeded that of 15-week-old WT mice [*t*(38) = 2.110, *p* = 0.0415, Figure 2C]. In the 21-week-old mice, the duration of time spent at the center [*t*(18) = 2.132, *p* = 0.0470, Figure 2E] and the percentage of distance traveled at the center [*t*(18) = 3.179, *p* = 0.0052, Figure 2D] of the open field were lower in Slit2-Tg mice than in 21-week-old WT mice, illustrating an anxiety-like state but an increased activity level in 21-week-old Slit2-Tg mice.

The number of vertical activities and total distance were significantly affected by genotype (vertical activities: *F*_1, 114_ = 16.440, *p* < 0.0001; total distance: *F*_1, 114_ = 25.467, *p* < 0.0001) and genotype × time interaction (vertical activities: *F*_2, 114_ = 7.559, *p* = 0.001; total distance: *F*_2, 114_ = 3.803, *p* = 0.025), with no significant time (vertical activities: *F*_2, 114_ = 0.561, *p* = 0.572; total distance: *F*_2, 114_ = 1.335, *p* = 0.267). *Post-hoc* comparisons revealed that the number of vertical activities made by 21-week-old WT mice was significantly lower than those of WT mice at 15 weeks old (*p* = 0.001, Figure 2F) and 9 weeks old (*p* = 0.007, Figure 2F). In addition, the total distance traveled in the open field was significantly increased in 21-week-old Slit2-Tg mice compared to 15-week-old Slit2-Tg mice (*p* = 0.007, Figure 2C) and 9-week-old Slit2-Tg mice (*p* = 0.005, Figure 2C). We found that the number of crossings was significantly affected only by genotype (genotype: *F*_1, 114_ = 5.625, *p* = 0.019; time: *F*_2, 114_ = 0.863, *P* = 0.425; genotype × time interaction: *F*_2, 114_ = 1.863, *p* = 0.160). Likewise, the total distance [*t*(18) = 12.04, *p* < 0.0001, Figure 2C], number of vertical activities [*t*(18) = 6.027, *p* < 0.0001, Figure 2F], and number of crossings [*t*(18) = 2.894, *p* = 0.0097, Figure 2G] was significantly increased in 21-week-old Slit2-Tg mice compared to WT mice at the same age. These results suggest that Slit2 overexpression causes increased anxiety-like behaviors and the persistence of anxiety-like behaviors with grown mice.

### Increased Anxiety-Like Behavior of Slit2-Tg Mice in the Elevated Plus Maze Test

**Figure 3:**
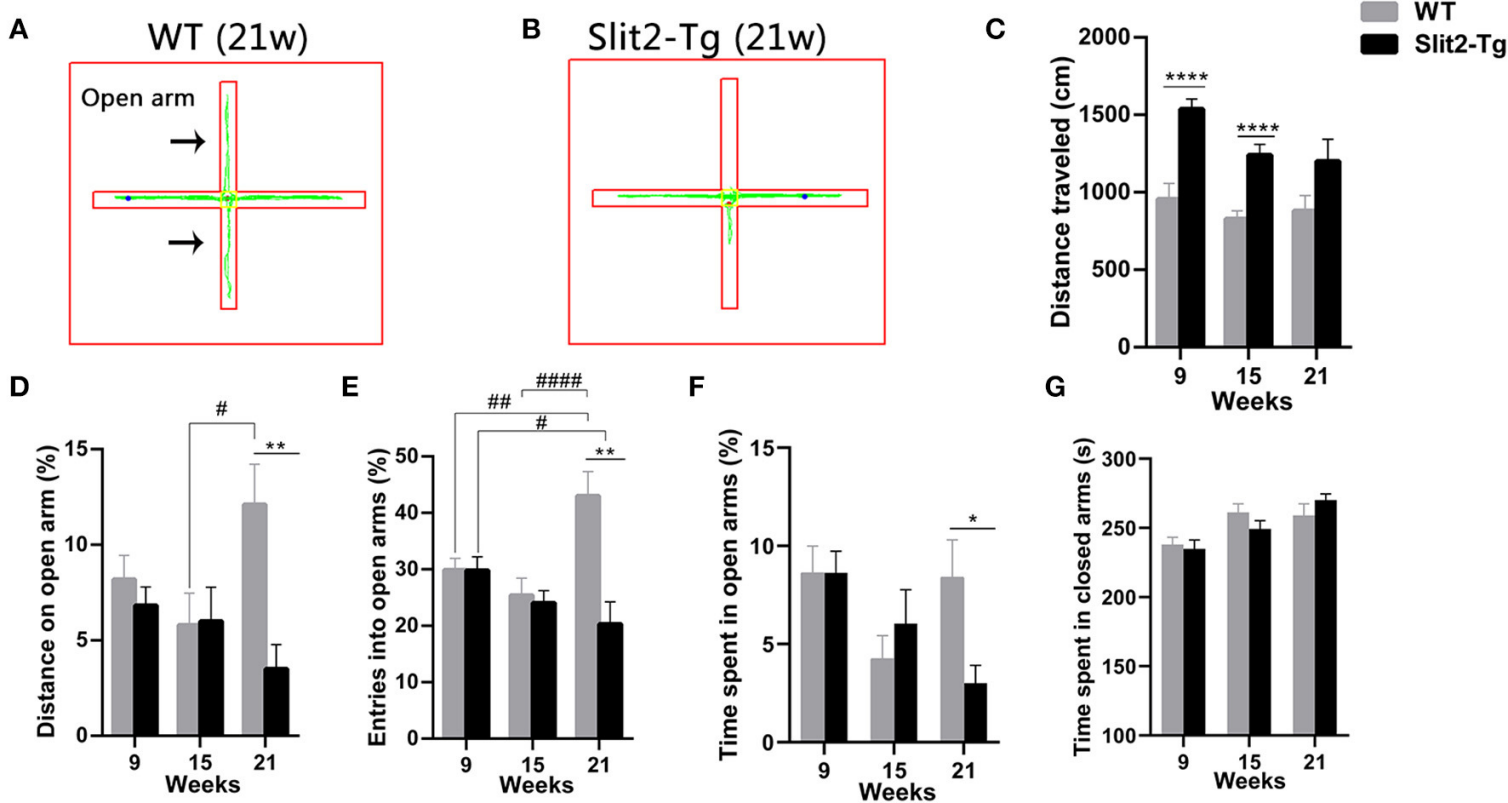
Slit2-Tg mice displayed increased anxiety-like behavior during the 5 min of the elevated plus maze test. **(A,B)** show the trajectories of mice in the elevated plus maze test. **(C)** Distance traveled. **(D)** Distance on the open arm (%). **(E)** Entry into open arms (%). **(F)** Time spent in the open arms (%). **(G)** Time spent in the closed arms (s). The elevated plus maze test showed decreased entry into the open arms in Slit2-Tg mice aged 21 weeks compared with wild-type (WT) mice of the same age. All data are expressed as means ± SEM, *p < 0.05, **p < 0.01, ****p < 0.0001 vs. WT mice. ^#^*p* < 0.05, ^##^*p* < 0.01, ^####^*p* < 0.0001 (Slit2-Tg mice: 9 weeks, *n* = 30; 15 weeks, *n* = 20; 21 weeks, *n* = 10; WT mice: 9 weeks, *n* = 30; 15 weeks, *n* = 20; 21 weeks, *n* = 10).

To further examine whether the overexpression of Slit2 results in abnormal behavior, anxiety-like behaviors were assessed by the elevated plus maze test. In the elevated plus maze test, the percentage of entries and the percentage of distance traveled in the open arm were affected by genotype (two-way ANOVA: entries, *F*_1, 114_ = 10.590, *p* = 0.001; distance traveled, *F*_1, 114_ = 5.980, *p* = 0.016) and genotype × time interaction (entries: F_2, 114_ = 6.805, *p* = 0.002; distance traveled: *F*_2, 114_ = 3.128, *p* = 0.048), with no significant time (entries: *F*_2, 114_ = 2.921, *p* = 0.058; distance traveled: *F*_2, 114_ = 0.887, *p* = 0.415). *Post hoc* multiple comparisons indicated that 21-week-old WT mice made a higher percentage of distance (*p* = 0.015, Figure 3D) and percentage of entries (*p* < 0.0001, Figure 3E) into the open arm than 15-week-old WT mice. In addition, the percentage of entries into the open arm in 21-week-old Slit2-Tg mice was lower than that in 9-week-old Slit2-Tg mice (*p* = 0.036, Figure 3E), while 21-week-old WT mice made a higher percentage of entries into the open arm than 9-week-old WT mice (*p* = 0.004, Figure 3E). The percentage of time spent in the open arm was significantly affected only by time (two-way ANOVA: time, *F*_2, 114_ = 4.005, *p* = 0.021; genotype, *F*_1, 114_ = 0.856, *p* = 0.357; genotype × time interaction, *F*_2, 114_ = 2.125, *p* = 0.124). As shown in Figure 3, the percentage of entries in the open arm [*t*(18) = 3.908, *p* = 0.0010, Figure 3E], the percentage of time spent in the open arms [*t*(18) = 2.547, *p* = 0.0202, Figure 3F], and the percentage of distance traveled in the open arm of the maze [*t*(18) = 3.474, *p* = 0.0027, Figure 3D] were significantly lower in 21-week-old Slit2-Tg mice than in 21-week-old WT mice, indicating increased anxiety-like behavior in 21-week-old Slit2-Tg mice.

Similarly, the time spent in the closed arm was significantly affected only by time (two-way ANOVA: time, *F*_2, 114_ = 9.326, *p* < 0.0001; genotype, *F*_1, 114_ = 0.059, *p* = 0.809; genotype × time interaction, *F*_2, 114_ = 1.068, *p* = 0.347). Twenty-one-week-old Slit2-Tg mice tended to spend more time in the closed arms than 21-week-old WT mice, but there was no significant difference [*t*(18) = 1.175, *p* = 0.2554, Figure 3G]. The total distance in the elevated test was significantly affected by genotype (*F*_1, 114_ = 29.596, *p* < 0.0001) and time (*F*_2, 114_ = 4.148, *p* = 0.018), with no significant genotype × time interaction (*F*_2, 114_ = 1.050, *p* = 0.353). In 21-week-old mice, no significant differences between Slit2-Tg and WT mice were found in total distance [*t*(18) = 1.861, *p* = 0.0791, Figure 3C]. The distance traveled by WT mice in the elevated test was larger than that by Slit2-Tg mice at 9 and 15 weeks of age [9 weeks: *t*(58) = 4.832, *p* < 0.0001; 15 weeks: *t*(38) = 4.774, *p* < 0.0001, Figure 3C].

### Impaired Neurons in the Hippocampus of Slit2-Tg Mice

**Figure 4:**
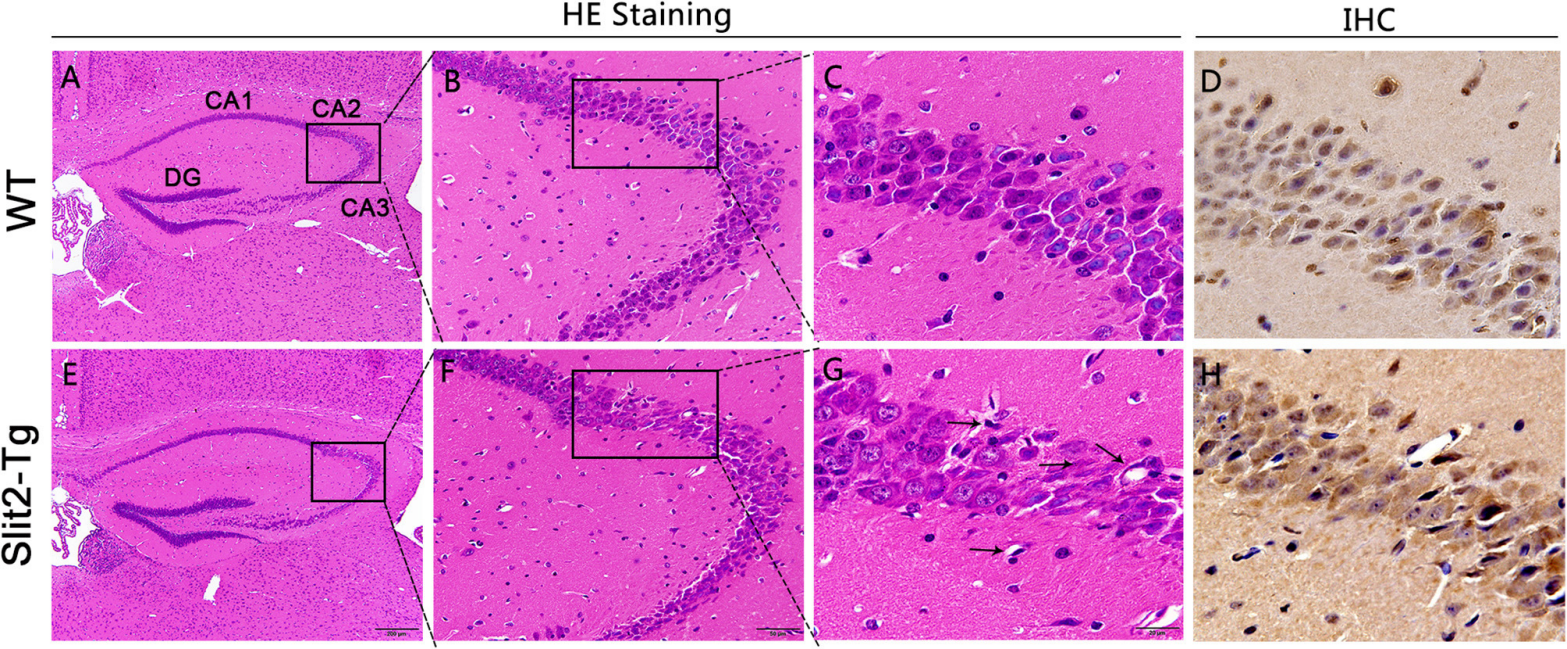
Effect of Slit2 overexpression on histopathological changes in the hippocampus of mice. The H&E and immunohistochemistry (IHC) staining were performed on paraffin section from brain of mice. **(A,E)** 10 × magnification; scale bar = 200 μm. **(B,F)** 40 × magnification; scale bar = 50 μm. **(C,G)** 100 × magnification; scale bar = 20 μm. **(D,H)** Immunohistochemical staining of Slit2 protein in hippocampal CA3 regions. 100 × magnification; scale bar = 20 μm. The necrotic neurons are shown with arrows.

H.E. staining was performed to detect the effects of Slit2 overexpression on morphology in the hippocampus. The CA2 and CA3 regions were marked using a black border. As shown in Figures 4B,C, the neurons in the hippocampal CA2 region of 23-week-old WT mice appeared intact and regularly arranged: round neurons or oval nuclei, clear nuclei, and compact structures. Nuclear pyknosis and neurons swelling were also not found in the 23-week-old WT mice. However, there were obvious histopathological and morphological changes in 23-week-old Slit2-Tg mice in the choroid plexus and the CA2 and CA3 regions of the hippocampus compared with 23-week-old WT mice (Figure 4G). The cell morphology in the hippocampus of Slit2-Tg mice were incomplete, the intercellular space were enlarged, the arrangement was disordered, and the number of cell layers is reduced. Neurons were sparse and absent in the 23-week-old Slit2-Tg mice. Additionally, some eccentric dispersion and expansion of neuronal bodies were also observed in the Slit2-Tg group (black arrow). These results illustrated that neurons in the hippocampal CA2 region of 23-week-old Slit2-Tg mice were partially impaired, although the neuron numbers were not significantly decreased in the CA1, CA3, or DG regions of the hippocampus. Moreover, we found that the choroid plexus in the lateral ventricle of Slit2-Tg mice was abnormal. The choroid plexus of 23-week-old WT mice was a tight, mono-cell layer covered with vessels (black border), while the choroid plexus of 23-week-old Slit2-Tg mice is damaged, in which there were gaps between the epithelial cell layer and the vessels (Supplementary Figure 2). Consistent with our previous report (Li et al., 2015), the choroid plexus of the 8–9-month-old Slit2-Tg mice was seriously damaged, further illustrating BBB damage. The immunohistochemistry results showed that Slit2 protein is expressed in the cytoplasm of neurons in the hippocampus of the mouse brain (Figures 4D,G), indicating that Slit2 protein is closely related to hippocampal neurons.

### Effect of Slit2 Overexpression on the Molecular Alteration in the Hippocampus of Mice

**Figure 5:**
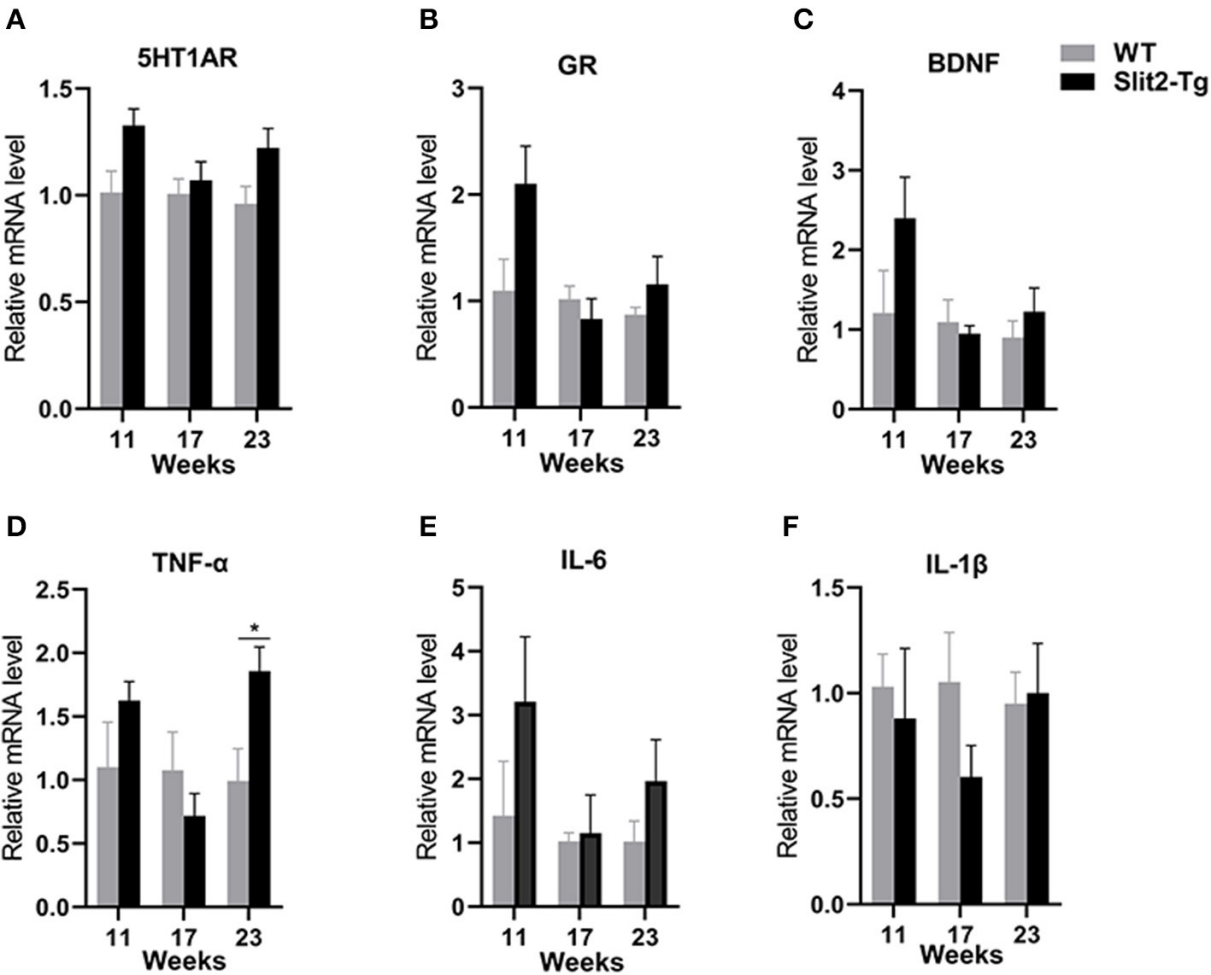
The messenger RNA (mRNA) expression levels of 5-HT1AR, BDNF, GR, TNF-α, IL-6, and IL-1β in the hippocampus of mice. Unpaired t-tests showed significant difference in TNF-α mRNA expression levels **(D)**. There were no significant difference in 5-HT1AR, GR, BDNF, IL-6, or IL-1β mRNA levels between Slit2-Tg and wild-type (WT) mice **(A–C, E, F)**. n = 4. *p < 0.05 vs. WT.

H&E staining of the hippocampus showed that hippocampal neurons were partially impaired in 21-week-old Slit2-Tg mice. Therefore, we next assessed the molecular alterations in the hippocampus of mice using reverse transcription PCR (RT-PCR). As shown in Figure 5, compared to the WT mice, there was no significant difference in Htr1a, BDNF, or glucocorticoid receptor (GR) mRNA levels between Slit2-Tg and WT mice at 11 or 17 weeks of age. It is notable that 5-hydroxytryptamine receptor 1A (5HT1AR), BDNF, and GR mRNA levels were higher in Slit2-Tg mice than in WT at 21 weeks, although this difference was not statistically significant. It has been reported that proinflammatory cytokines are closely related to depression-like behaviors. We also detected the mRNA expression of IL-1β, IL-6, and TNF-α in the hippocampus of mice. As shown in Figure 5, based on the mRNA expression of TNF-α, IL-1β, and IL-6 in the hippocampus, there were no significant differences between WT and Slit2-Tg mice at 11 or 17 weeks of age. Significant upregulation of TNF-α mRNA expression was observed in 23-week-old Slit2-Tg mice compared with 23-week-old WT mice (Figure 5D). It is noteworthy that the mRNA expression of IL-1β and IL-6 tended to increase in the 23-week-old Slit2-Tg mice compared with WT mice of the same age, although this difference was not statistically significant.

## Discussion

In this study, we evaluated several behavioral features of mice at different ages. First, the locomotor activity of Slit2-Tg mice was increased from 9 to 21 weeks old, while the WT mice exhibited reduced locomotor activity. It has been reported that the peripheral and central nervous systems of WT mice will exhibit similar changes, such as decreased motor function with increasing age (Shoji et al., 2016). Second, 21-week-old Slit2-Tg mice exhibited increased anxiety- and depression-like behavior compared with WT mice. Third, the hippocampal neurons in 23-week-old Slit2-Tg mice were partially impaired. Fourth, only the level of TNF-α was increased in the hippocampus of 23-week-old Slit2-Tg mice, indicating that mild inflammation caused by overexpression of Slit2 could underlie anxiety- and depression-like behavior. Overall, we discovered that 23-week-old Slit2-Tg mice display several anxiety- and depression-like behavioral abnormalities, including decreased weight, anhedonia, impaired hippocampal morphology, and molecular alterations in the brain. Additionally, 23-week-old Slit2-Tg mice possessed a damaged choroid plexus phenotype that has also been identified in Slit2-Tg mice at 8–9 months of age. These results suggest that 23-week-old Slit2-Tg mice have anxiety- and depression-like behaviors, which may be used as a spontaneous animal model of anxiety and depression.

The secreted glycoprotein Slit2, as an axon guiding factor, plays critical roles in the development of the central nervous system (Brose et al., 1999). Thus, abnormalities in the Slit/Robo pathway are observed in these neuropsychiatric disorders. There is increasing evidence that suggest Slit2 is associated with some neuropsychiatric disorders, including MDD (Huls et al., 2020), AD (Li et al., 2015), Parkinson's disease (PD) (Lin and Isacson, 2006), TLE (Fang et al., 2010), and ASD (Perez et al., 2016; Gorker et al., 2018). Depression and anxiety are common psychiatric disorders, and their pathogenesis is not well-understood. Thus, studying the molecular mechanisms of depression/anxiety is crucial. An epigenome-wide association study (EWAS) of MDD patients found that altered methylation in the Slit2 locus is associated with late-life depression (Huls et al., 2020). However, there is little clinical evidence that Slit2 is associated with depression, and there is lack of direct experimental and experimental evidence to support this view. The Slit2-Tg mice were transgenic mice constructed with the background of C57BL/6J. In this study, we performed a battery of behavioral tests on Slit2-Tg and C57BL/6J mice at 9, 15, and 21 weeks of age to better detect changes in the behavioral characteristics of adult Slit2-Tg mice. The present assessment results revealed that body weight was significantly decreased in the 21-week-old Slit2-Tg compared to WT mice of the same age. Depression could cause weight loss. Therefore, we assessed whether Slit2-Tg mice displayed depression-like behavior changes. Depression-like behaviors in Slit2-Tg mice were assessed based on the index of sucrose preference, which reflected anhedonia. In sucrose preference test (SPT) and compared with the 21-week-old WT mice, the index of sucrose preference was significantly reduced in Slit2-Tg mice of the same age, illustrating that the 21-week-old Slit2-Tg mice exhibited depression-like behavior. Likewise, the increased immobility time of 21-week-old Slit2-Tg mice in the forced swim test suggests that adult Slit2-Tg mice showed a tendency for depression. Thus, these data further supplemented previous studies from animal experiments and show that Slit2 is associated with depression. In particular, these results indicated that the overexpression of Slit2 could be the potential molecular mechanism of depression.

Most depression is accompanied by anxiety. Compared with 21-week-old WT mice, the percentage of time in the open arms, distance on open arms and the percentage of entries into open arms of the maze, and the percentage of time spent at the center and the percentage of distance traveled at the center of the open field were significantly decreased in the Slit2-Tg mice of the same age, illustrating that the adult Slit2-Tg mice exhibited increased anxiety-like behavior. However, Slit2-Tg and WT mice showed significant differences in crossing numbers, vertical activity, and total distance traveled in the open field test at the age of 21-week-old. It should be noted that the results of the forced swim and the tail suspension tests were inconsistent. Slit2-Tg mice exhibited depression-like behavior but displayed hyperactivity in the tail suspension test. Consistent with Matsuda's report (Matsuda et al., 2016), the results of the tail suspension and the forced swim tests may not simply reflect changes in depression-like behavior. The hyperactivity of Slit2-Tg mice was observed in the tail suspension test, possibly due to anxiety. Studies have reported that mouse activity is related to the integrity of brain structure (Ten et al., 2004). Our previous studies have shown increased permeability of the BBB and AD-like alterations in 8–9-month-old Slit2-Tg mice. In addition, Han and Geng (2011) found larger lateral ventricles and increased brain vessel density and permeability in Slit2-Tg mice at 8–12 week of age. The abnormally increased activity and hyperactive phenotype of Slit2-Tg mice may be due to brain anatomical defects. The 21-week-old Slit2-Tg mice exhibited hyperactivity, which may also be due to lower body weight or anxiety (Sakai et al., 2016). Overall, these results suggest depression-/anxiety-like behavior alterations in adult Slit2 transgenic mice, although these results might have been influenced by measurement error.

Patients with depression are often accompanied by anxiety, and some animal experiment results also show the coexistence of depression and anxiety (Brady and Kendall, 1992; Tiller, 2013; Gao et al., 2018). To our knowledge, this is the first experimental report that found that Slit2 is associated with depression and anxiety, and Slit2 could be playing critical roles in depression and anxiety.

Our data show that 21-week-old Slit2-Tg mice exhibit symptoms similar to those observed in patients with major depression or anxiety disorders. However, at present, there are no specific biomarkers that can predict patient response to antidepressants (Zeier et al., 2018). Patients with depression and anxiety patients have abnormal hippocampal activity (Johnston et al., 2015). The hippocampus has been suggested to play important roles in long-term memory storage and memory consolidation (Squire et al., 2004). In animal models, depression is accompanied by a decrease in the number of hippocampal neurons (Yuan et al., 2015). The hippocampus is a key location for regulating mood and anxiety (Matsushita et al., 2019). Intensive research has implicated the hippocampus in anxiety and depression (Sheline et al., 2003; Bannerman et al., 2004; Duman and Monteggia, 2006). We also evaluated the effects of Slit2 overexpression on brain structure and function to further understand the significance of Slit2 protein in neuropsychiatric disorders. From H&E staining, we found morphological changes in the hippocampus in 23-week-old Slit2-Tg mice, including a small number with an absent hippocampal neurons or impaired choroid plexus. The immunohistochemistry results showed that Slit2 protein is expressed in the cytoplasm of hippocampal neurons, indicating that Slit2 protein is closely related to hippocampal neurons. A study revealed that Slit mRNAs were widely expressed in the hippocampus of the primate brain with modest regional preference (Sasaki et al., 2020). Our data in combination with our previous studies have shown that increased permeability of the BBB and damaged neurons caused by overexpression of Slit2 could be underlying causes of depression and anxiety.

There is increasing evidence that the monoamine neurotransmitter serotonin is related to the etiology and course of the anxiety disorders and MDD (Albert et al., 2012; Albert and Benkelfat, 2013). The serotonin (5-hydroxytryptamine; 5-HT) autoreceptor 5-HT receptor 1A (5HT1AR) is the most widely distributed 5-HT receptor subtype. Studies have demonstrated that the 5HT1A receptors are involved in the pathogenesis of depression (Samuels et al., 2016) Moreover, symptoms of anxiety and depression are always accompanied by changes in the expression of 5-HT1A receptors (Xiang et al., 2017) In this study, it should be noted that regardless of age, the expression level of 5-HT1A receptors in Slit2-Tg mice tended to increase compared with that in WT mice, although these differences were not statistically significant. This may be because Slit2 overexpression upregulates the expression of 5-HT receptor 1A. Evidence supports negative feedback from the 5-HT1A receptor in the synthesis and release of serotonin, which may be one of the reasons why Slit2-Tg mice exhibit anxiety- and depression-like behaviors (Frey et al., 2008) This hypothesis seems to be consistent with the result of a study reporting that transgenic mice expressing human mutant LRRK2 G2019S exhibited anxiety-/depression-like behavior, and a significant increase in 5-HT1A receptor level was detected in the hippocampus of transgenic mice (Lim et al., 2018). It has also been reported that the mRNA of 5-HT receptor 1A is significantly increased in patients with schizophrenia (Mohammadi et al., 2018). It has also been reported that autopsies of MDD patients found elevated levels of BDNF protein in the brain (Krishnan et al., 2007). Depression is often accompanied by excessive activation of the hypothalamic–pituitary–adrenal axis (HPA) axis, which leads to excessive secretion of glucocorticoids (GCs) and activation of glucocorticoid receptors (GRs) (Meyer et al., 2014; Lou et al., 2018). In the present study, there was no statistical difference in the expression levels of BDNF and GR between Slit2-Tg mice and WT mice.

An increasing amount of data also suggests that inflammation has an important role in the pathophysiology of depression and anxiety (Miller et al., 2013; Felger, 2018). It has been reported that proinflammatory cytokines are closely related to depression-like behaviors (Raison and Miller, 2011). Our data show that the mRNA expression of TNF-α, IL-1β, and IL-6 was increased in the hippocampus of 23-week-old Slit2-Tg mice, although the changes in IL-1β and IL-6 mRNA expression were not statistically significant. A somewhat related study showed that depression and anxiety disorders may be caused by chronic activation of the immune response (Sutin et al., 2010; Miller et al., 2013). Peripheral cytokines access the brain through the BBB (Dantzer et al., 2008). The blood–CSF barrier (BCSFB) is formed by tight junctions between the choroid plexus epithelial cells, and the epithelial cells produce cerebrospinal fluid (CSF), which is crucial for the regulation of the BBB (Kaur et al., 2016). In this study, the choroid plexus of 23-week-old Slit2-Tg mice is damaged, indicating that peripheral small molecules may cross the BBB and enter the brain parenchyma. All of these results demonstrated that depression- and anxiety-like phenotypes were observed in 23-week-old Slit2-Tg mice, which might be partially due to elevated proinflammatory factors.

In conclusion, depression and anxiety, as chronic and recurrent emotional disorders, cause a huge economic burden to society. Due to this, there is an urgent need to better understand anxiety and depression pathophysiology. Studies have shown that KO mice lacking G-protein-coupled monoaminergic receptors (e.g., 5-HT1B, 5-HT1A, and 5-HT4 receptors) provided the spontaneous model of depressive disorders, which is the main target of antidepressant drugs (Gardier et al., 2009). Compared with 5-HT KO mice, although no significant changes in monoamine transmitters were observed in Slit2-Tg mice, the mice were able to show coexistence of anxiety and depression. Slit2-Tg mice, thus, display both anxiety and anhedonia, making them a potent animal model in the treatment of forms of depression comorbidly expressed with anxiety. Slit2 is an exocrine protein and has also been described as an axon repellent. Slit2 binds to their transmembrane receptor, Robo, to prevent inappropriate midline crossing by axons in the CNS. In addition, Slit2 is also involved in the distribution, migration, and branching of neuron cells and plays an important role in the development of the central nervous system. The role of Slit2 in various CNS disease models has gradually attracted attention. Previous studies have shown that a larger lateral ventricle area, increased number of structurally incomplete vessels in the brain, and more permeability in the blood vessels have been observed in Slit2-Tg mice (Han and Geng, 2011). The tight junctions in the choroid plexus were also destroyed in Slit2-Tg mice (Han and Geng, 2011). Our results demonstrated that 23-week-old Slit2-Tg mice exhibited choroid plexus and hippocampus abnormalities and increased the TNF-α mRNA. These alterations may bring about depression- and anxiety-like behaviors in 23-week-old mature adult Slit2-Tg mice and may provide a spontaneous model for studying mental disorders. The lack of enough mechanism-related result is the limitation of this study. Slit2 is expressed by neurons in the normal adult brain in rats and the human brain. In the case of brain damage, Slit2 is expressed by glial cells in addition to neurons, such as in glioma or epilepsy (Fang et al., 2010; Jin et al., 2016). This implicates distinct functions of Slit2 in the adult brain. In our study, the results of immunohistochemistry showed that Slit2 was expressed in pyramidal cells in the hippocampus of the mouse brain. In addition, Li et al. used RT quantitative PCR (RT-qPCR) analysis to show the overexpression of Slit2 in the brain of the Slit2-Tg mice, compared with that in WT mice (Li et al., 2018). However, this still does not confirm how Slit2 overexpression in specific tissues or cell types in the brain might lead to the behavioral changes. The relationship between Slit2 and depression- and anxiety-like behavior needs to be further investigated and will be the focus of future work in our lab. Future studies will be required to understand the alterations of the BBB and abnormal behavior observed in adult Slit2-Tg mice and to identify the molecular mechanisms underlying these changes.

## Data Availability Statement

The original contributions presented in the study are included in the article/Supplementary Material, further inquiries can be directed to the corresponding author/s.

## Ethics Statement

The animal study was reviewed and approved by Animal Ethics Committee of Guangdong Pharmaceutical University.

## Author Contributions

GH and SW: data analysis and interpretation. GH, CL, JF, JY, QC, XZ, YH, and HL: investigation. LW, SW, and GH: methodology. LW, WL, JL, and HL: resources. GH: writing-original draft. SW, LW, AY, and JL: manuscript critical revisions.

## Conflict of Interest

The authors declare that the research was conducted in the absence of any commercial or financial relationships that could be construed as a potential conflict of interest.

## Abbreviations

Slit2-Tg: Slit2-overexpressing transgenic
H&E: hematoxylin–eosin staining
CNS: central nervous system
Robo: roundabout
AD: Alzheimer's disease
ASD: autism spectrum disorder
TLE: temporal lobe epilepsy
MDD: major depressive disorder
CSF: cerebrospinal fluid
BBB: blood–brain barrier
CUMS: chronic unpredictable mild stress
BDNF: brain-derived neurotrophic factor
GR: glucocorticoid receptor
5HT1AR: 5-hydroxytryptamine receptor 1A
IL-1β: interleukin-1β
IL-6: interleukin-6
TNF-α: tumor necrosis factor α
EWAS: epigenome-wide association study
PD: Parkinson disease.
