# Supplementary figures and images for: Depression-/Anxiety-Like Behavior Alterations in Adult Slit2 Transgenic Mice
Source: Front Behav Neurosci. 2021 Feb 5;14:622257. doi: 10.3389/fnbeh.2020.622257 (PMC7892588; doi:10.3389/fnbeh.2020.622257)

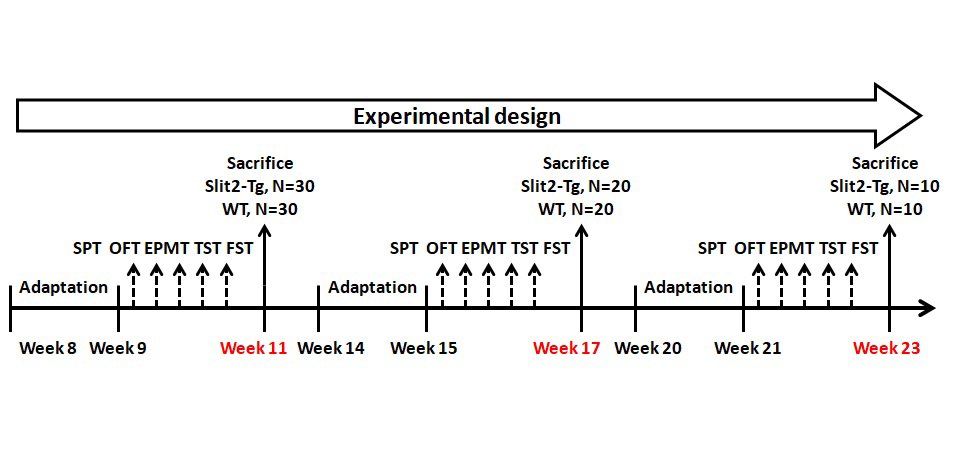

Supplement: Supplementary file 1 [file Image_1.TIF]

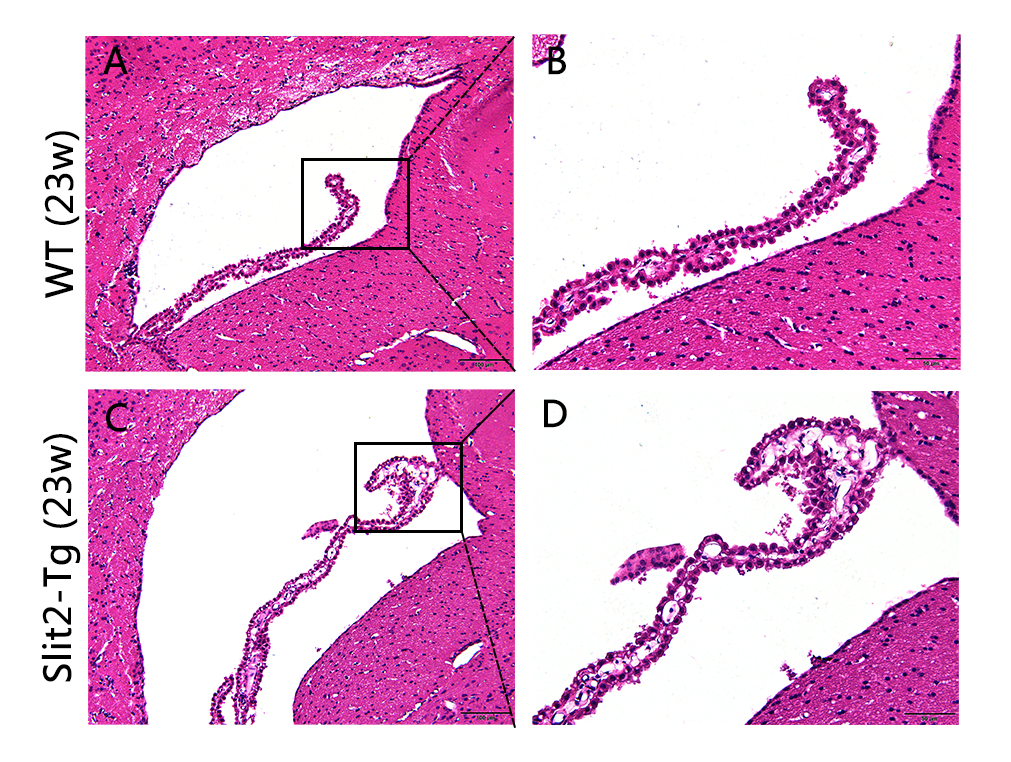

Supplement: Supplementary file 2 [file Image_2.TIF]

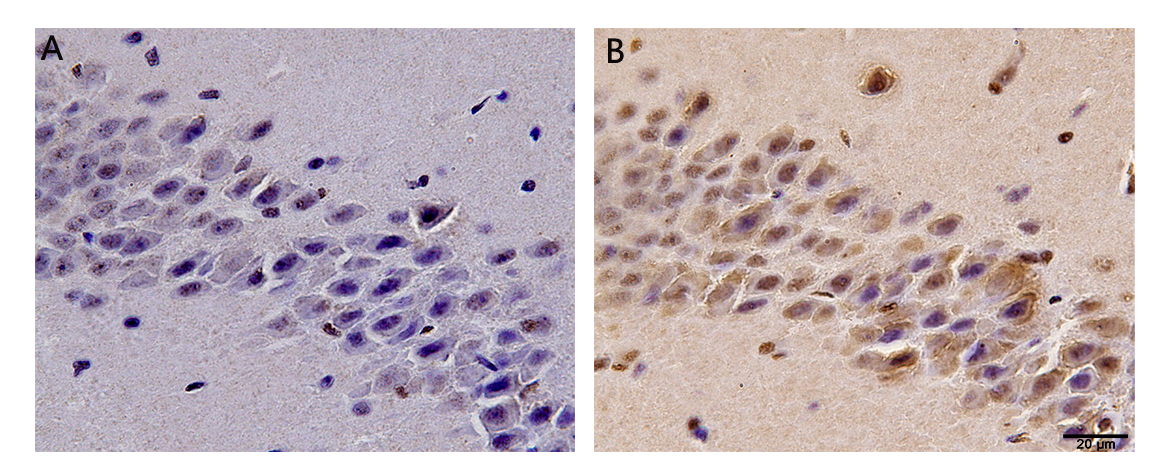

Supplement: Supplementary file 3 [file Image_3.tif]

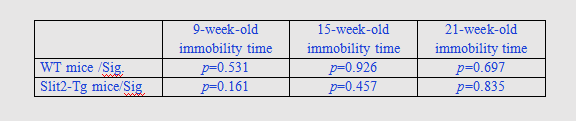

Supplement: Supplementary file 5 [file Image_5.tif]
